# Ethyl 2-(3,4-dimethyl-5,5-dioxo-1*H*,4*H*-benzo[*e*]pyrazolo­[4,3-*c*][1,2]thia­zin-1-yl)acetate

**DOI:** 10.1107/S1600536812039797

**Published:** 2012-09-26

**Authors:** Sana Aslam, Hamid Latif Siddiqui, Matloob Ahmad, Muhammad Zia-ur-Rehman, Masood Parvez

**Affiliations:** a Institute of Chemistry, University of the Punjab, Lahore 54590, Pakistan; b Department of Chemistry, Government College University, Faisalabad 38000, Pakistan; c Applied Chemistry Research Center, PCSIR Laboratories Complex, Lahore 54600, Pakistan; d Department of Chemistry, University of Calgary, 2500 University Drive NW, Calgary, Alberta, Canada T2N 1N4

## Abstract

In the title mol­ecule, C_15_H_17_N_3_O_4_S, the heterocyclic thia­zine ring adopts a twist-boat conformation, which differs from that in related compounds, with adjacent S and C atoms displaced by 0.981 (4) and 0.413 (5) Å, respectively, on the same side of the mean plane formed by the remaining ring atoms. The mean plane of the benzene ring makes a dihedral angle of 23.43 (14)° with the mean plane of the pyrazole ring. In the crystal, mol­ecules are connected by weak C—H⋯O hydrogen bonds to form a three-dimensional network. The H atoms of the methyl group attached to the pyrazole ring were refined over six sites with equal occupancies.

## Related literature

For background literature and crystal structures of related pyrazolo­benzothia­zine derivatives, see: Aslam *et al.* (2012 ▶); Ahmad *et al.* (2012 ▶). For the Cambridge Structural Database, see: Allen (2002 ▶).
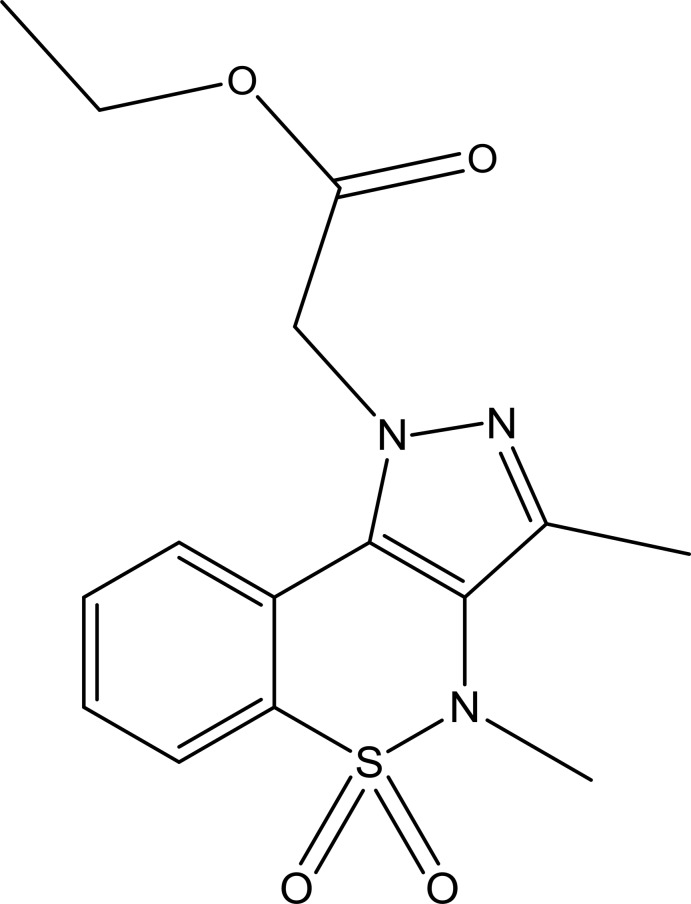


## Experimental

### 

#### Crystal data

C_15_H_17_N_3_O_4_S
*M* *_r_* = 335.38
Monoclinic, 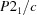

*a* = 8.3027 (2) Å
*b* = 8.5915 (3) Å
*c* = 22.3476 (7) Å
β = 90.674 (2)°
*V* = 1594.00 (8) Å^3^
*Z* = 4
Mo *K*α radiation
μ = 0.23 mm^−1^
*T* = 173 K
0.20 × 0.18 × 0.16 mm

#### Data collection

Nonius KappaCCD diffractometer
Absorption correction: multi-scan (*SORTAV*; Blessing, 1997 ▶) *T*_min_ = 0.956, *T*_max_ = 0.965
15091 measured reflections
3576 independent reflections
2820 reflections with *I* > 2σ(*I*)
*R*_int_ = 0.039

#### Refinement

*R*[*F* ^2^ > 2σ(*F* ^2^)] = 0.056
*wR*(*F* ^2^) = 0.127
*S* = 1.10
3576 reflections
210 parameters
H-atom parameters constrained
Δρ_max_ = 0.33 e Å^−3^
Δρ_min_ = −0.45 e Å^−3^

### 

Data collection: *COLLECT* (Nonius, 1998 ▶); cell refinement: *DENZO* (Otwinowski & Minor, 1997 ▶); data reduction: *SCALEPACK* (Otwinowski & Minor, 1997 ▶); program(s) used to solve structure: *SHELXS97* (Sheldrick, 2008 ▶); program(s) used to refine structure: *SHELXL97* (Sheldrick, 2008 ▶); molecular graphics: *ORTEP-3 for Windows* (Farrugia, 1997 ▶); software used to prepare material for publication: *SHELXL97*.

## Supplementary Material

Crystal structure: contains datablock(s) global, I. DOI: 10.1107/S1600536812039797/lh5533sup1.cif

Structure factors: contains datablock(s) I. DOI: 10.1107/S1600536812039797/lh5533Isup2.hkl

Supplementary material file. DOI: 10.1107/S1600536812039797/lh5533Isup3.cml

Additional supplementary materials:  crystallographic information; 3D view; checkCIF report

## Figures and Tables

**Table 1:** Hydrogen-bond geometry (Å, °)

*D*—H⋯*A*	*D*—H	H⋯*A*	*D*⋯*A*	*D*—H⋯*A*
C12—H12*A*⋯O2^i^	0.99	2.43	3.338 (3)	152
C12—H12*B*⋯O1^ii^	0.99	2.29	3.255 (3)	165
C4—H4⋯O2^iii^	0.95	2.58	3.290 (3)	132
C10—H10*C*⋯O4^iv^	0.98	2.51	3.369 (4)	147
C14—H14*B*⋯O1^v^	0.99	2.55	3.424 (4)	147

Symmetry codes: (i) 
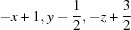
; (ii) 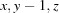; (iii) 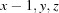; (iv) 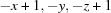; (v) 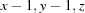.

**Fig. 1.**
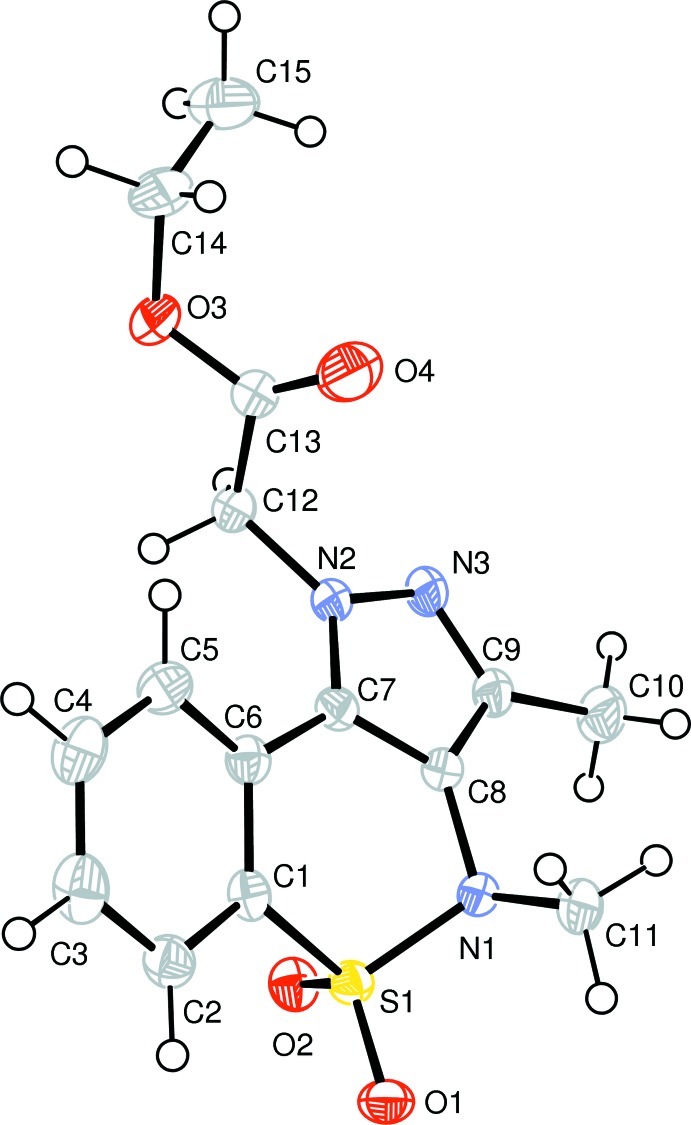
The molecular structure of the title compound with displacement ellipsoids drawn at the 30% probability level. H atoms are presented as small spheres of an arbitrary radius.

**Fig. 2.**
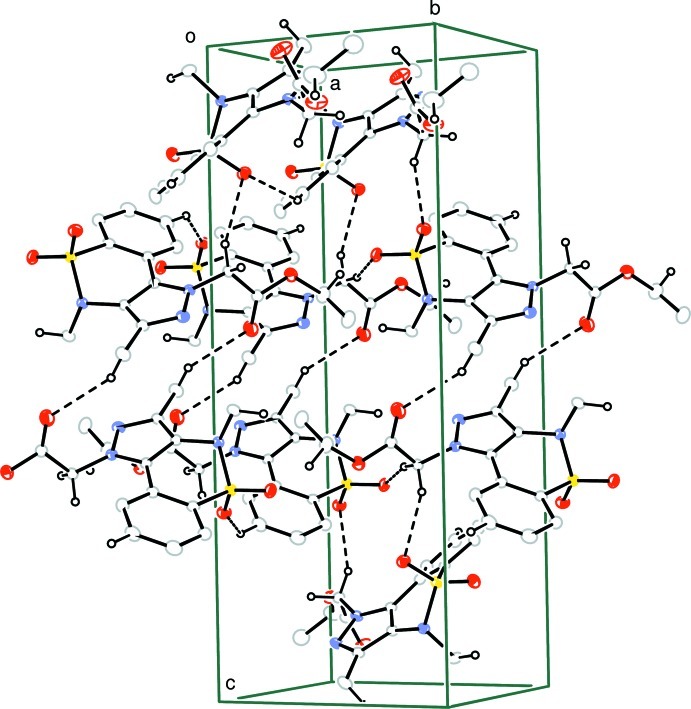
The weak hydrogen bonds (dashed lines) in the crystal structure of the title compound. H atoms non-participating in hydrogen bonds are omitted for clarity.

## supplementary crystallographic information

### Comment

Continuing our research on hybrid pyrazolobenzothiazine derivatives based on pyrazole and benzothiazine nuclei which are well known for their wide range of biological activities (Ahmad *et al.*, 2012) we have synthsized the title compound which is a novel product wherein the ethylecatate has been substituted at N1 of the pyrazole ring instead of the usual N2 position. A search of the Cambridge Structural Database (Allen, 2002; CSD Version 5.33) revealed eleven structures with substituents at the N2 position and only one structure with substituents at both N1 and N2 positions and no pyrazolobenzothiazine derivative with a substituent at the N1 position. We report the synthesis and crystal structure of the title compound in this article.

The bond distances and angles in the title compound (Fig. 1) agree very well with those reported in closely related structures (Aslam *et al.*, 2012; Ahmad *et al.*, 2012). The heterocyclic thiazine ring adopts a twist-boat conformation with atoms S1 and C1 displaced by 0.981 (4) and 0.413 (5) Å, respectively, on the same side from the mean plane formed by the remaining ring atoms (N1/C6–C8 atoms). The mean-plane of the benzene ring C1–C6 makes a dihedral angle 23.43 (14)° with the mean-plane of the pyrazole ring (N2/N3/C7/C8/C9). The acetate group (O3/O4/C12/C13/C14) is essentially planar (rmsd 0.031 Å) and its mean-plane is oriented at 82.4 (2)° with respect to the pyrazole ring.

The crystal structure is stabilized by weak intermolecular C—H···O hydrogen bonds resulting in a three-dimensional network (Fig. 2 and Table 1).

### Experimental

A mixture of 3,4-dimethyl-2,4-dihydropyrazolo[4,3-*c*][1,2]benzothiazine 5,5-dioxide (5.0 g, 0.020 moles), anhydrous potassium carbonate (3.31 g, 0.024 moles), ethyl chloroacetate (2.94 g, 0.024 moles) and acetonitrile (30 ml) was refluxed for 10 h followed by the removal of solvent under vacuum. The residue obtained was washed with cold water to get the title compound as a white crystalline product. Transparent crystals suitable for X-ray crystallographic studies were grown from a CHCl_3_ solution at room temperature by slow evaporation.

### Refinement

All H atoms were positioned geometrically and refined using a riding model, with C—H = 0.95–0.99 Å and *U*_iso_(H) = 1.2*U*_eq_(C) or 1.5*U*_eq_(C_methyl_).

### Crystal data

**Table:**

C_15_H_17_N_3_O_4_S	*F*(000) = 704
*M**_r_* = 335.38	*D*_x_ = 1.398 Mg m^−^^3^
Monoclinic, *P*2_1_/*c*	Mo *K*α radiation, λ = 0.71073 Å
Hall symbol: -P 2ybc	Cell parameters from 3460 reflections
*a* = 8.3027 (2) Å	θ = 1.0–27.5°
*b* = 8.5915 (3) Å	µ = 0.23 mm^−^^1^
*c* = 22.3476 (7) Å	*T* = 173 K
β = 90.674 (2)°	Block, colourless
*V* = 1594.00 (8) Å^3^	0.20 × 0.18 × 0.16 mm
*Z* = 4	

### Data collection

**Table:**

Nonius KappaCCD diffractometer	3576 independent reflections
Radiation source: fine-focus sealed tube	2820 reflections with *I* > 2σ(*I*)
Graphite monochromator	*R*_int_ = 0.039
ω and φ scans	θ_max_ = 27.5°, θ_min_ = 3.0°
Absorption correction: multi-scan (*SORTAV*; Blessing, 1997)	*h* = −10→10
*T*_min_ = 0.956, *T*_max_ = 0.965	*k* = −11→11
15091 measured reflections	*l* = −28→28

### Refinement

**Table:**

Refinement on *F*^2^	Primary atom site location: structure-invariant direct methods
Least-squares matrix: full	Secondary atom site location: difference Fourier map
*R*[*F*^2^ > 2σ(*F*^2^)] = 0.056	Hydrogen site location: inferred from neighbouring sites
*wR*(*F*^2^) = 0.127	H-atom parameters constrained
*S* = 1.10	*w* = 1/[σ^2^(*F*_o_^2^) + (0.0235*P*)^2^ + 2.7353*P*] where *P* = (*F*_o_^2^ + 2*F*_c_^2^)/3
3576 reflections	(Δ/σ)_max_ < 0.001
210 parameters	Δρ_max_ = 0.33 e Å^−^^3^
0 restraints	Δρ_min_ = −0.45 e Å^−^^3^

### Special details

**Table:**

Geometry. All e.s.d.'s (except the e.s.d. in the dihedral angle between two l.s. planes) are estimated using the full covariance matrix. The cell e.s.d.'s are taken into account individually in the estimation of e.s.d.'s in distances, angles and torsion angles; correlations between e.s.d.'s in cell parameters are only used when they are defined by crystal symmetry. An approximate (isotropic) treatment of cell e.s.d.'s is used for estimating e.s.d.'s involving l.s. planes.
Refinement. Refinement of *F*^2^ against ALL reflections. The weighted *R*-factor *wR* and goodness of fit *S* are based on *F*^2^, conventional *R*-factors *R* are based on *F*, with *F* set to zero for negative *F*^2^. The threshold expression of *F*^2^ > σ(*F*^2^) is used only for calculating *R*-factors(gt) *etc*. and is not relevant to the choice of reflections for refinement. *R*-factors based on *F*^2^ are statistically about twice as large as those based on *F*, and *R*-factors based on ALL data will be even larger.

### Fractional atomic coordinates and isotropic or equivalent isotropic displacement parameters (Å^2^)

**Table:**

	*x*	*y*	*z*	*U*_iso_*/*U*_eq_	Occ. (<1)
S1	0.60768 (7)	0.28409 (8)	0.67513 (3)	0.02293 (16)	
O1	0.6208 (2)	0.4498 (2)	0.67696 (8)	0.0301 (4)	
O2	0.7203 (2)	0.1922 (2)	0.70884 (8)	0.0295 (4)	
O3	0.1309 (3)	−0.4262 (3)	0.64119 (9)	0.0435 (6)	
O4	0.1716 (3)	−0.2669 (3)	0.56356 (10)	0.0537 (7)	
N1	0.6194 (2)	0.2279 (3)	0.60491 (9)	0.0233 (5)	
N2	0.4504 (3)	−0.1506 (3)	0.61898 (9)	0.0251 (5)	
N3	0.5677 (3)	−0.1867 (3)	0.57904 (10)	0.0296 (5)	
C1	0.4117 (3)	0.2290 (3)	0.69663 (11)	0.0231 (5)	
C2	0.3265 (3)	0.3239 (3)	0.73520 (12)	0.0289 (6)	
H2	0.3708	0.4194	0.7490	0.035*	
C3	0.1753 (3)	0.2761 (4)	0.75310 (13)	0.0365 (7)	
H3	0.1166	0.3378	0.7806	0.044*	
C4	0.1088 (3)	0.1396 (4)	0.73138 (13)	0.0352 (7)	
H4	0.0046	0.1088	0.7438	0.042*	
C5	0.1925 (3)	0.0476 (3)	0.69173 (12)	0.0300 (6)	
H5	0.1438	−0.0440	0.6760	0.036*	
C6	0.3483 (3)	0.0884 (3)	0.67452 (11)	0.0237 (5)	
C7	0.4492 (3)	0.0034 (3)	0.63269 (11)	0.0230 (5)	
C8	0.5724 (3)	0.0676 (3)	0.59991 (11)	0.0231 (5)	
C9	0.6426 (3)	−0.0531 (3)	0.56744 (11)	0.0273 (6)	
C10	0.7810 (4)	−0.0464 (4)	0.52519 (14)	0.0400 (7)	
H10A	0.8020	−0.1507	0.5094	0.060*	0.50
H10B	0.8771	−0.0083	0.5465	0.060*	0.50
H10C	0.7547	0.0243	0.4921	0.060*	0.50
H10D	0.8205	0.0609	0.5226	0.060*	0.50
H10E	0.7454	−0.0815	0.4855	0.060*	0.50
H10F	0.8679	−0.1141	0.5399	0.060*	0.50
C11	0.5531 (4)	0.3344 (3)	0.55871 (12)	0.0354 (7)	
H11A	0.5831	0.2970	0.5189	0.053*	
H11B	0.5968	0.4391	0.5650	0.053*	
H11C	0.4355	0.3375	0.5616	0.053*	
C12	0.3618 (3)	−0.2766 (3)	0.64581 (11)	0.0268 (6)	
H12A	0.3318	−0.2465	0.6870	0.032*	
H12B	0.4325	−0.3692	0.6487	0.032*	
C13	0.2108 (3)	−0.3195 (3)	0.61103 (12)	0.0289 (6)	
C14	−0.0120 (4)	−0.4933 (5)	0.61234 (15)	0.0498 (9)	
H14A	−0.0681	−0.4125	0.5884	0.060*	
H14B	−0.0871	−0.5311	0.6432	0.060*	
C15	0.0347 (4)	−0.6243 (4)	0.57295 (16)	0.0516 (9)	
H15A	−0.0622	−0.6712	0.5552	0.077*	
H15B	0.0930	−0.7027	0.5966	0.077*	
H15C	0.1042	−0.5855	0.5411	0.077*	

### Atomic displacement parameters (Å^2^)

**Table:**

	*U*^11^	*U*^22^	*U*^33^	*U*^12^	*U*^13^	*U*^23^
S1	0.0218 (3)	0.0242 (3)	0.0228 (3)	−0.0016 (3)	0.0000 (2)	−0.0012 (3)
O1	0.0321 (10)	0.0244 (10)	0.0339 (10)	−0.0043 (8)	0.0013 (8)	−0.0057 (8)
O2	0.0242 (9)	0.0367 (11)	0.0276 (9)	0.0017 (8)	−0.0034 (7)	0.0017 (8)
O3	0.0440 (12)	0.0537 (15)	0.0330 (11)	−0.0269 (11)	0.0023 (9)	0.0034 (10)
O4	0.0597 (15)	0.0542 (16)	0.0466 (14)	−0.0224 (12)	−0.0224 (12)	0.0196 (12)
N1	0.0263 (11)	0.0227 (11)	0.0210 (10)	−0.0037 (9)	0.0032 (8)	−0.0002 (9)
N2	0.0313 (11)	0.0237 (12)	0.0204 (10)	−0.0042 (9)	0.0045 (9)	−0.0025 (9)
N3	0.0361 (12)	0.0298 (13)	0.0231 (11)	−0.0019 (10)	0.0093 (9)	−0.0039 (10)
C1	0.0232 (12)	0.0264 (13)	0.0198 (12)	0.0043 (10)	0.0011 (9)	0.0015 (10)
C2	0.0317 (14)	0.0263 (15)	0.0287 (14)	0.0032 (11)	0.0035 (11)	−0.0015 (11)
C3	0.0338 (15)	0.0379 (17)	0.0381 (16)	0.0142 (13)	0.0132 (12)	0.0002 (14)
C4	0.0221 (13)	0.0428 (18)	0.0408 (16)	0.0041 (12)	0.0078 (11)	0.0069 (14)
C5	0.0249 (13)	0.0311 (15)	0.0342 (15)	−0.0011 (12)	0.0007 (11)	0.0007 (12)
C6	0.0220 (12)	0.0270 (14)	0.0219 (12)	0.0015 (10)	−0.0008 (10)	0.0019 (10)
C7	0.0234 (12)	0.0256 (13)	0.0200 (12)	−0.0018 (10)	−0.0012 (10)	−0.0006 (10)
C8	0.0249 (12)	0.0238 (13)	0.0206 (12)	−0.0034 (10)	0.0010 (10)	0.0000 (10)
C9	0.0327 (14)	0.0275 (14)	0.0217 (13)	−0.0016 (12)	0.0066 (11)	−0.0007 (11)
C10	0.0475 (18)	0.0366 (17)	0.0362 (16)	−0.0020 (14)	0.0200 (14)	−0.0044 (14)
C11	0.0515 (18)	0.0303 (16)	0.0244 (14)	−0.0047 (14)	−0.0019 (12)	0.0074 (12)
C12	0.0332 (14)	0.0236 (13)	0.0234 (13)	−0.0036 (11)	0.0003 (11)	0.0028 (11)
C13	0.0323 (14)	0.0284 (15)	0.0260 (14)	−0.0043 (12)	0.0016 (11)	−0.0004 (11)
C14	0.0363 (17)	0.063 (2)	0.050 (2)	−0.0272 (17)	0.0035 (15)	−0.0070 (18)
C15	0.052 (2)	0.047 (2)	0.055 (2)	−0.0165 (17)	−0.0085 (17)	−0.0043 (18)

### Geometric parameters (Å, º)

**Table:**

S1—O1	1.429 (2)	C6—C7	1.458 (3)
S1—O2	1.4312 (19)	C7—C8	1.381 (3)
S1—N1	1.646 (2)	C8—C9	1.397 (4)
S1—C1	1.767 (3)	C9—C10	1.497 (4)
O3—C13	1.321 (3)	C10—H10A	0.9800
O3—C14	1.462 (3)	C10—H10B	0.9800
O4—C13	1.195 (3)	C10—H10C	0.9800
N1—C8	1.435 (3)	C10—H10D	0.9800
N1—C11	1.481 (3)	C10—H10E	0.9800
N2—C7	1.358 (3)	C10—H10F	0.9800
N2—N3	1.365 (3)	C11—H11A	0.9800
N2—C12	1.443 (3)	C11—H11B	0.9800
N3—C9	1.333 (3)	C11—H11C	0.9800
C1—C2	1.387 (4)	C12—C13	1.513 (4)
C1—C6	1.405 (4)	C12—H12A	0.9900
C2—C3	1.384 (4)	C12—H12B	0.9900
C2—H2	0.9500	C14—C15	1.483 (5)
C3—C4	1.381 (4)	C14—H14A	0.9900
C3—H3	0.9500	C14—H14B	0.9900
C4—C5	1.381 (4)	C15—H15A	0.9800
C4—H4	0.9500	C15—H15B	0.9800
C5—C6	1.399 (4)	C15—H15C	0.9800
C5—H5	0.9500		
			
O1—S1—O2	119.04 (12)	H10A—C10—H10C	109.5
O1—S1—N1	108.32 (12)	H10B—C10—H10C	109.5
O2—S1—N1	107.12 (11)	C9—C10—H10D	109.5
O1—S1—C1	109.24 (12)	H10A—C10—H10D	141.1
O2—S1—C1	107.91 (12)	H10B—C10—H10D	56.3
N1—S1—C1	104.22 (11)	H10C—C10—H10D	56.3
C13—O3—C14	117.3 (2)	C9—C10—H10E	109.5
C8—N1—C11	116.1 (2)	H10A—C10—H10E	56.3
C8—N1—S1	109.67 (17)	H10B—C10—H10E	141.1
C11—N1—S1	117.25 (18)	H10C—C10—H10E	56.3
C7—N2—N3	112.1 (2)	H10D—C10—H10E	109.5
C7—N2—C12	129.2 (2)	C9—C10—H10F	109.5
N3—N2—C12	118.2 (2)	H10A—C10—H10F	56.3
C9—N3—N2	105.6 (2)	H10B—C10—H10F	56.3
C2—C1—C6	122.2 (2)	H10C—C10—H10F	141.1
C2—C1—S1	119.3 (2)	H10D—C10—H10F	109.5
C6—C1—S1	118.50 (19)	H10E—C10—H10F	109.5
C3—C2—C1	118.4 (3)	N1—C11—H11A	109.5
C3—C2—H2	120.8	N1—C11—H11B	109.5
C1—C2—H2	120.8	H11A—C11—H11B	109.5
C4—C3—C2	120.7 (3)	N1—C11—H11C	109.5
C4—C3—H3	119.7	H11A—C11—H11C	109.5
C2—C3—H3	119.7	H11B—C11—H11C	109.5
C5—C4—C3	120.6 (3)	N2—C12—C13	113.1 (2)
C5—C4—H4	119.7	N2—C12—H12A	109.0
C3—C4—H4	119.7	C13—C12—H12A	109.0
C4—C5—C6	120.5 (3)	N2—C12—H12B	109.0
C4—C5—H5	119.8	C13—C12—H12B	109.0
C6—C5—H5	119.8	H12A—C12—H12B	107.8
C5—C6—C1	117.5 (2)	O4—C13—O3	125.6 (3)
C5—C6—C7	126.2 (2)	O4—C13—C12	125.4 (3)
C1—C6—C7	116.1 (2)	O3—C13—C12	109.0 (2)
N2—C7—C8	105.2 (2)	O3—C14—C15	110.2 (3)
N2—C7—C6	129.7 (2)	O3—C14—H14A	109.6
C8—C7—C6	125.1 (2)	C15—C14—H14A	109.6
C7—C8—C9	107.2 (2)	O3—C14—H14B	109.6
C7—C8—N1	123.0 (2)	C15—C14—H14B	109.6
C9—C8—N1	129.6 (2)	H14A—C14—H14B	108.1
N3—C9—C8	109.9 (2)	C14—C15—H15A	109.5
N3—C9—C10	121.3 (3)	C14—C15—H15B	109.5
C8—C9—C10	128.8 (3)	H15A—C15—H15B	109.5
C9—C10—H10A	109.5	C14—C15—H15C	109.5
C9—C10—H10B	109.5	H15A—C15—H15C	109.5
H10A—C10—H10B	109.5	H15B—C15—H15C	109.5
C9—C10—H10C	109.5		
			
O1—S1—N1—C8	−167.65 (16)	N3—N2—C7—C6	−177.6 (2)
O2—S1—N1—C8	62.78 (19)	C12—N2—C7—C6	−6.1 (4)
C1—S1—N1—C8	−51.42 (19)	C5—C6—C7—N2	−27.8 (4)
O1—S1—N1—C11	−32.5 (2)	C1—C6—C7—N2	156.1 (3)
O2—S1—N1—C11	−162.02 (19)	C5—C6—C7—C8	155.4 (3)
C1—S1—N1—C11	83.8 (2)	C1—C6—C7—C8	−20.7 (4)
C7—N2—N3—C9	0.2 (3)	N2—C7—C8—C9	0.2 (3)
C12—N2—N3—C9	−172.2 (2)	C6—C7—C8—C9	177.6 (2)
O1—S1—C1—C2	−28.1 (2)	N2—C7—C8—N1	−175.4 (2)
O2—S1—C1—C2	102.6 (2)	C6—C7—C8—N1	2.1 (4)
N1—S1—C1—C2	−143.7 (2)	C11—N1—C8—C7	−98.0 (3)
O1—S1—C1—C6	152.79 (19)	S1—N1—C8—C7	37.7 (3)
O2—S1—C1—C6	−76.4 (2)	C11—N1—C8—C9	87.5 (3)
N1—S1—C1—C6	37.2 (2)	S1—N1—C8—C9	−136.7 (3)
C6—C1—C2—C3	1.2 (4)	N2—N3—C9—C8	−0.1 (3)
S1—C1—C2—C3	−177.9 (2)	N2—N3—C9—C10	179.6 (2)
C1—C2—C3—C4	−2.2 (4)	C7—C8—C9—N3	0.0 (3)
C2—C3—C4—C5	0.5 (4)	N1—C8—C9—N3	175.1 (2)
C3—C4—C5—C6	2.3 (4)	C7—C8—C9—C10	−179.7 (3)
C4—C5—C6—C1	−3.2 (4)	N1—C8—C9—C10	−4.6 (5)
C4—C5—C6—C7	−179.2 (3)	C7—N2—C12—C13	97.8 (3)
C2—C1—C6—C5	1.5 (4)	N3—N2—C12—C13	−91.2 (3)
S1—C1—C6—C5	−179.50 (19)	C14—O3—C13—O4	4.4 (5)
C2—C1—C6—C7	177.9 (2)	C14—O3—C13—C12	−174.7 (3)
S1—C1—C6—C7	−3.1 (3)	N2—C12—C13—O4	6.1 (4)
N3—N2—C7—C8	−0.2 (3)	N2—C12—C13—O3	−174.8 (2)
C12—N2—C7—C8	171.2 (2)	C13—O3—C14—C15	85.1 (4)

### Hydrogen-bond geometry (Å, º)

**Table:**

*D*—H···*A*	*D*—H	H···*A*	*D*···*A*	*D*—H···*A*
C12—H12*A*···O2^i^	0.99	2.43	3.338 (3)	152
C12—H12*B*···O1^ii^	0.99	2.29	3.255 (3)	165
C4—H4···O2^iii^	0.95	2.58	3.290 (3)	132
C10—H10*C*···O4^iv^	0.98	2.51	3.369 (4)	147
C14—H14*B*···O1^v^	0.99	2.55	3.424 (4)	147
C11—H11*B*···O1	0.98	2.51	2.872 (3)	102

Symmetry codes: (i) −*x*+1, *y*−1/2, −*z*+3/2; (ii) *x*, *y*−1, *z*; (iii) *x*−1, *y*, *z*; (iv) −*x*+1, −*y*, −*z*+1; (v) *x*−1, *y*−1, *z*.
